# Adaptive response to chronic mild ethanol stress involves ROS, sirtuins and changes in chromosome dosage in wine yeasts

**DOI:** 10.18632/oncotarget.8673

**Published:** 2016-04-10

**Authors:** Jagoda Adamczyk, Anna Deregowska, Marek Skoneczny, Adrianna Skoneczna, Aleksandra Kwiatkowska, Leszek Potocki, Ewa Rawska, Sylwia Pabian, Jakub Kaplan, Anna Lewinska, Maciej Wnuk

**Affiliations:** ^1^ Department of Genetics, University of Rzeszow, Rejtana, Rzeszow, Poland; ^2^ Postgraduate School of Molecular Medicine, Medical University of Warsaw, Warsaw, Poland; ^3^ Department of Genetics, Institute of Biochemistry and Biophysics, Polish Academy of Sciences, Warsaw, Poland; ^4^ Laboratory of Mutagenesis and DNA Repair, Institute of Biochemistry and Biophysics, Polish Academy of Sciences, Warsaw, Poland; ^5^ Department of Biochemistry and Cell Biology, University of Rzeszow, Rzeszow, Poland

**Keywords:** wine yeasts, genome, array-CGH, ethanol, sirtuins, Gerotarget

## Abstract

Industrial yeast strains of economic importance used in winemaking and beer production are genomically diverse and subjected to harsh environmental conditions during fermentation. In the present study, we investigated wine yeast adaptation to chronic mild alcohol stress when cells were cultured for 100 generations in the presence of non-cytotoxic ethanol concentration. Ethanol-induced reactive oxygen species (ROS) and superoxide signals promoted growth rate during passages that was accompanied by increased expression of sirtuin proteins, Sir1, Sir2 and Sir3, and DNA-binding transcription regulator Rap1. Genome-wide array-CGH analysis revealed that yeast genome was shaped during passages. The gains of chromosomes I, III and VI and significant changes in the gene copy number in nine functional gene categories involved in metabolic processes and stress responses were observed. Ethanol-mediated gains of *YRF1* and *CUP1* genes were the most accented. Ethanol also induced nucleolus fragmentation that confirms that nucleolus is a stress sensor in yeasts. Taken together, we postulate that wine yeasts of different origin may adapt to mild alcohol stress by shifts in intracellular redox state promoting growth capacity, upregulation of key regulators of longevity, namely sirtuins and changes in the dosage of genes involved in the telomere maintenance and ion detoxification.

## INTRODUCTION

Ethanol stress is one of the major environmental stresses generated during microbe-based industrial fermentations, e.g., beer production or winemaking that may affect their performance [1, 2]. The elucidation of the mechanisms of ethanol adaptive responses and tolerance in industrial yeast strains of *Saccharomyces cerevisiae* species is of fundamental scientific interest as well as of economic importance due to heavy demand for alternative energy sources, namely renewable biofuels such as ethanol [3].

As ethanol increases the fluidity and permeability of the plasma membrane affecting the functions of membrane proteins and cell transport that, in turn, may compromise yeast cell physiology, ethanol-induced cell response is primarily based on the changes in membrane composition preventing membrane fluidization and promoting its stabilization [1, 4], e.g., by increased levels of unsaturated fatty acids (UFAs) [5] or ergosterol [6]. Moreover, the addition of selected amino acids and inositol may confer some resistance to ethanol by membrane stabilization [7–10]. Trehalose and heat shock proteins (HSPs) may also play a protective role against ethanol-mediated aggregation of denaturated and misfolded proteins [11, 12]. More recently, it has been postulated and rebutted that alcohol sensitive ring/PHD finger 1 protein Asr1p is required for tolerance to media containing alcohol [13, 14]. Asr1p has been reported to constitutively shuttle between nucleus and cytoplasm but accumulate in the nucleus upon exposure to ethanol, 2-propanol or 1-butanol [13]. However, Asr1-based signaling pathway has been suggested to not be critical for the response to ethanol and brewing sake and making wine [14]. As ethanol exerted pleiotropic effects on cells, multiple and divergent signaling pathways may take part in the ethanol stress response [11, 15, 16]. According to DNA microarray analysis, 30-min ethanol stress (7%) induced a transient transcriptional response that mainly involves the environmental stress response (ESR) genes and the stress gene family [15]. This may suggest that ethanol response exhibits some functional overlap with other stress responses *via* transcription factors Msn2p and Msn4p of general stress response system [17–19].

In the present study, long-term effects of non-cytotoxic concentration of ethanol were investigated using eight commercially available wine yeast strains. We found that ethanol-mediated adaptation during passages is based on ROS and superoxide signaling, sirtuins and changes in the gene copy number as judged by genome-wide array-based comparative genomic hybridization (array-CGH).

## RESULTS

### Diploid nature and *S. cerevisiae*-like karyotype profiles of wine yeasts

**Figure 1:**
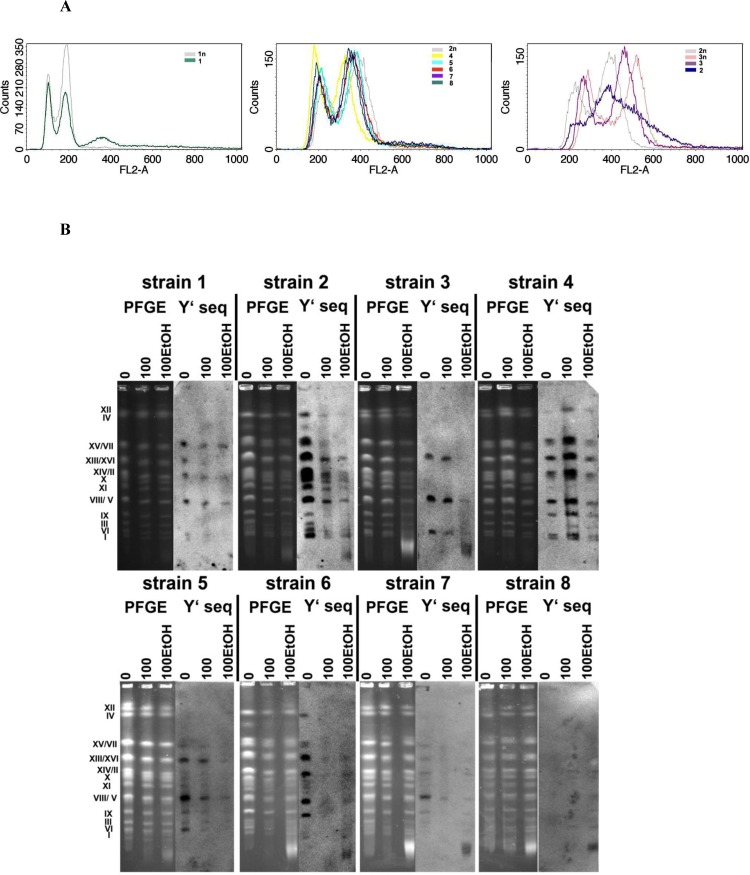
The ploidy analysis (A) and generation- and ethanol-mediated karyotype profiles and telomere status (B) **A.** Fluorescence-activated cell sorting (FACS)-based analysis of DNA content of wine strains (1 to 8). Representative histograms are shown. Haploid, diploid and triploid reference strains are also presented. **B.** Electrophoretic karyotyping (PFGE separation) of wine yeast strains and the presence of telomeric Y’ sequences. Lanes 0, control conditions; lanes 100, 100 generations; lanes 100EtOH, 100 generations in the presence of 5% EtOH. PFGE, genomic DNA after PFGE separation.

As wine yeast strains were purchased from multiple suppliers (Table 1), first, we have characterized their ploidy state and karyotype profiles (Figure 1).

Except of haploid strain 1 and triploid strain 3, all other strains were found to be diploid (Figure 1A). The histograms of strains from 4 to 8 were almost identical to the histogram of diploid reference yeast strain (Figure 1A). The histogram of strain 2 is more ambiguous but also shows some features of the diploid reference strain histogram (Figure 1A). The *S. cerevisiae*-like chromosome patterns were revealed in all analyzed strains (Figure 1B). In general, the chromosome number of analyzed strains is 16 (Figure 1B). However, some additional bands can also be observed, e.g., an additional band between chromosomes IV and VII was shown for strains 2 and 6 (Figure 1B) that is a characteristic feature of *S. bayanus* karyotype [20]. Perhaps, some of analyzed strains may be considered as hybrids between *S. cerevisiae* and *S. bayanus* (*S. cerevisiae var. bayanus*) that is also in agreement with information provided by the suppliers.

**Table 1:** Wine yeast strains used in this study

No.	Trade name	Supplier
1	Portwein	Biowin
2	Bordeaux	Biowin
3	Tokay	Biowin
4	Tokay 22	Zamojscy
5	Fermivin	Biowin
6	Aromatic Wine Complex	Spirit ferm
7	PDM Maurivin	Mauri
8	PRIMEUR Maurivin	Mauri

According to the suppliers' information provided, all strains were classified as *Saccharomyces cerevisiae* or *S. cerevisiae var. bayanus*.

### Y’ telomeric sequences are lost during passages

Wine yeast strains were then cultured for 100 generations in the presence and in the absence of 5% ethanol being a non-cytotoxic concentration (spot assay, data not shown). No gross numerical or structural abnormalities such as translocations were observed during passages (Figure 1B). The strain-specific pattern of Y’ telomeric sequences was shown (Figure 1B). In general, the loss of Y’ telomeric sequences was observed during passages (Figure 1B). The effect was more evident when cells were cultured in the presence of ethanol (Figure 1B). Moreover, ethanol-mediated fragmentation of Y’ telomeric sequences was also shown (strains 2, 3, 6, 7 and 8) (Figure 1B).

### ROS and superoxide signals promote growth during passages

**Figure 2:**
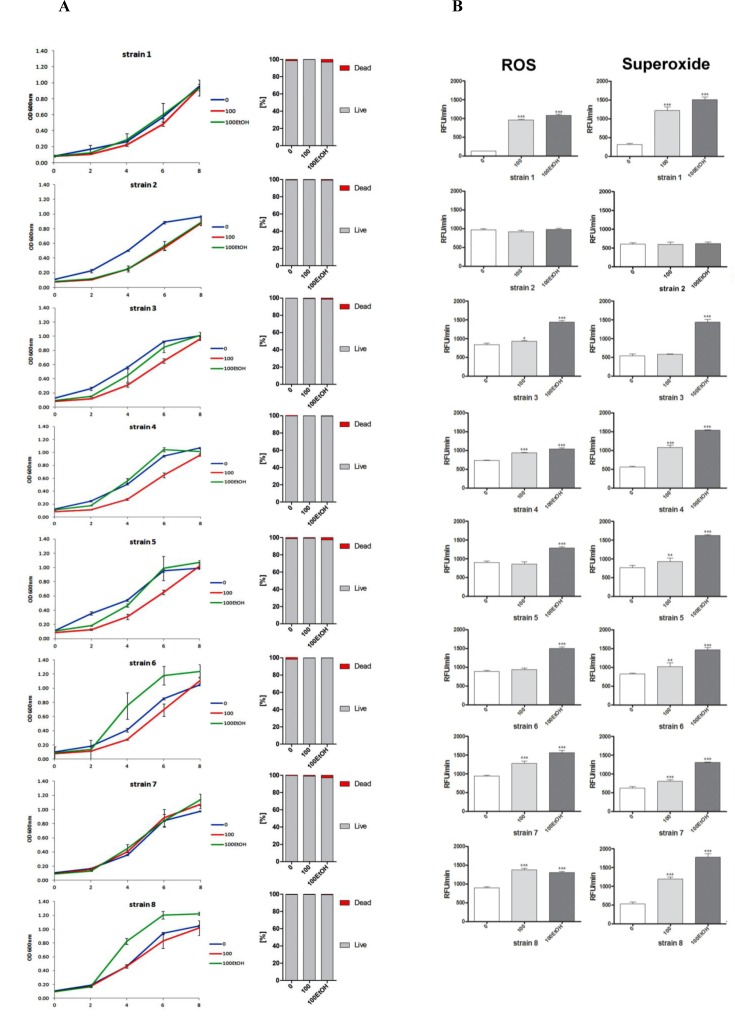
Generation- and ethanol-mediated changes in the growth rate and viability (A), and the production of total reactive oxygen species (ROS) and superoxide (B) **A.** Yeast growth was monitored turbidimetrically at 600 nm in a microplate reader every 2 h during a 8 h. Bars indicate SD, *n* = 6. Cell viability was estimated with a LIVE/DEAD^®^ Yeast Viability Kit using the standard protocol according to the manufacturer's instructions. The percentage of live and dead cells is shown, *n* = 200. **B.** Reactive oxygen species (ROS) production and superoxide were assessed using H_2_DCF-DA and dihydroethidium fluorogenic probes, respectively. The results are presented as relative fluorescence units per minute (RFU/min). Bars indicate SD, *n* = 4. ^***^p < 0.001, ^**^p < 0.01, ^*^p < 0.05 compared to the standard growth conditions (ANOVA and Dunnett's *a posteriori* test).

Except of strains 1 and 7, the growth rate was affected during passages both with and without ethanol (Figure 2A, left).

Generation- and ethanol-mediated acceleration and delay of growth was observed (Figure 2A, left). The effect was strain-dependent (Figure 2A, left). Decreased growth rate did not result from cell death as cell viability was not compromised (Figure 2A, right). Diminished growth was correlated with unaffected total reactive oxygen species (ROS) and superoxide production that was the most evidently observed in strain 2 (Figure 2B). When total ROS production was increased approximately from 50 to 70%, the growth rate was comparable to growth rate at control conditions (strains 3, 4, 5 and 7) or even augmented (strains 6 and 8) (Figure 2B, left). However, ethanol-induced acceleration of growth rate did not reflect the levels of ROS generated, e.g., ethanol caused approximately 9-fold increase in ROS production in strain 1 but this did not result in the growth acceleration. The growth rate of strain 1 during passages with ethanol was comparable to growth rate at control conditions. Perhaps, ROS acted at narrow concentration window to promote/improve growth during passages (Figure 2B, left). This also may be true for superoxide production (Figure 2B, right).

### Wine strains vary in redox state and susceptibility to DNA breaks

**Figure 3:**
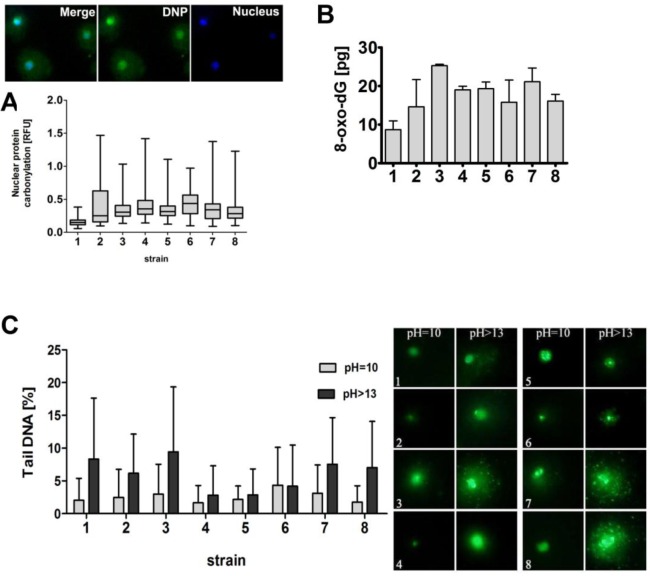
The susceptibility of wine yeast strains to oxidative damage to biomolecules and DNA breaks **A.** Nuclear protein carbonylation. Co-localization analysis of nuclear DNA (blue) with DNP-immuno-signals (green) was performed. DNA was visualized using DAPI staining (blue). Fluorescence intensity of DNP signals was analyzed using ImageJ software. The integrated fluorescence density is presented in relative fluorescence units (RFUs). Box and whisker plots are shown, *n* = 200. **B.** The level of 8-hydroxy-2′-deoxyguanosine (8-oxo-dG) was analyzed using ELISA-based assay. Bars indicate SD, *n* = 3. **C.** DNA double-strand breaks (DSBs) and DNA single-strand breaks (SSBs) were assessed using neutral and alkaline comet assay, respectively. As a DNA damage marker, the % tail DNA was used. Bars indicate SD, *n* = 150. The typical micrographs are shown (right). DNA was visualized using YOYO-1 staining (green).

In general, analyzed strains were characterized by differences in ROS production of 30% in control growth conditions (Figure 2B, left). However, ROS production in strain 1 was lowered approximately 6- to 8-fold compared to other strains in untreated controls (Figure 2B, left). Superoxide production in strain 1 was also lowered approximately 2- to 3-fold compared to other strains in untreated controls (Figure 2B, right). Thus, we decided to investigate more comprehensively the intracellular redox equilibrium of wine yeasts in standard growth conditions (Figure 3).

We analyzed nuclear protein carbonylation using imaging cytometry and found that strain 1 with the lowest production of ROS was also characterized by the lowest level of nuclear protein carbonylation (Figure 3A). Analogically, strain 1 was the least affected by oxidative DNA damage (Figure 3B). We also evaluated if variable ROS production may also account for altered genetic stability of wine yeasts (Figure 3C). However, increased ROS production did not correlate with elevated DNA double-strand breaks (DSBs) and DNA single-strand breaks (SSBs) in strain 1 (Figure 3C). The generation of DSBs and SSBs was comparable in strains 1 and 2 with the lowest and the highest production of ROS, respectively (Figure 2B, left and Figure 3).

### Changes in the chromosome status and gene dosage during passages

**Figure 4:**
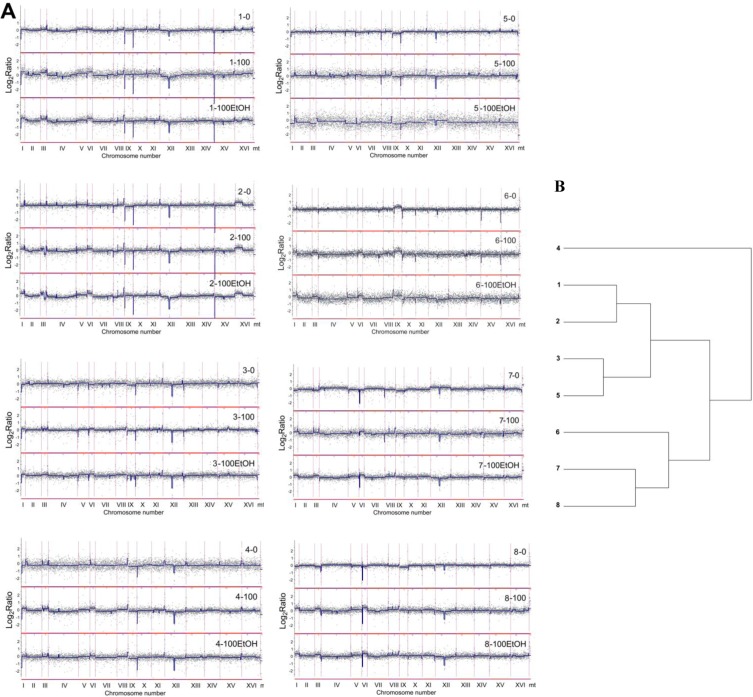
Analysis of the variability in the gene copy number of wine strains (1 to 8) using array-CGH **A.** Array-CGH profiles are shown. Each grey dot represents the value of the log_2_ ratio for an individual gene. Blue lines were provided to emphasize the most accented differences (DNA losses and gains). 0, control conditions; 100, 100 generations; 100EtOH, 100 generations in the presence of 5% EtOH. **B.** The relatedness of strains analyzed using cluster analysis. Similarity tree is shown.

**Figure 5:**
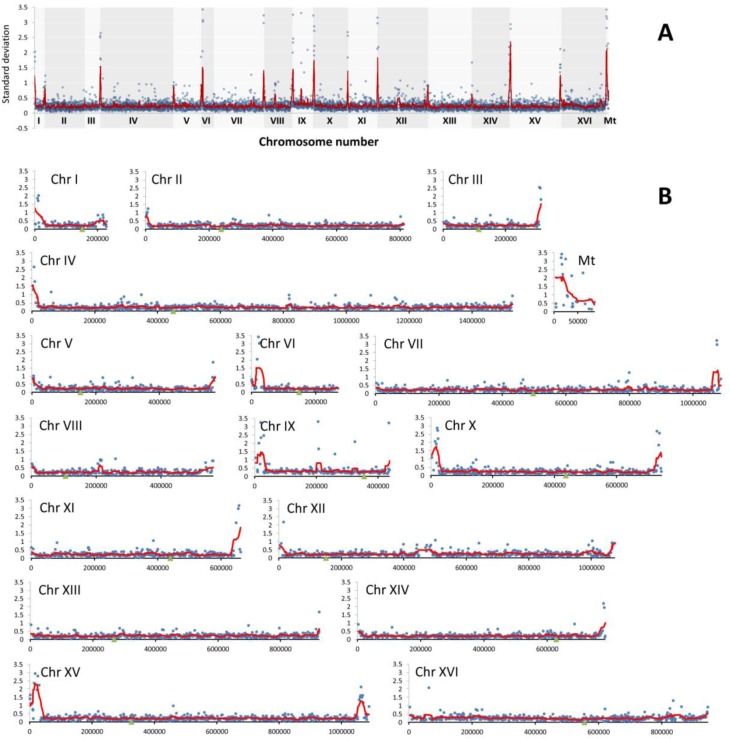
The divergence of relative abundance of genes as determined by array-CGH represented by standard deviation (SD) of log_**2**_ ratio values for each gene in wine strains **A.** The summary plot for the whole genome. **B.** Individual plots for each chromosome. Blue dots indicate the SD values for individual genes, the red line denotes the smoother trend calculated by moving average of SD values to expose the genome regions of higher log_2_ ratio divergence and green triangles indicate centromere position.

The genome of wine strains was characterized using array-based comparative genomic hybridization (array-CGH) (Figure 4 and Figure 5).

**Figure 6:**
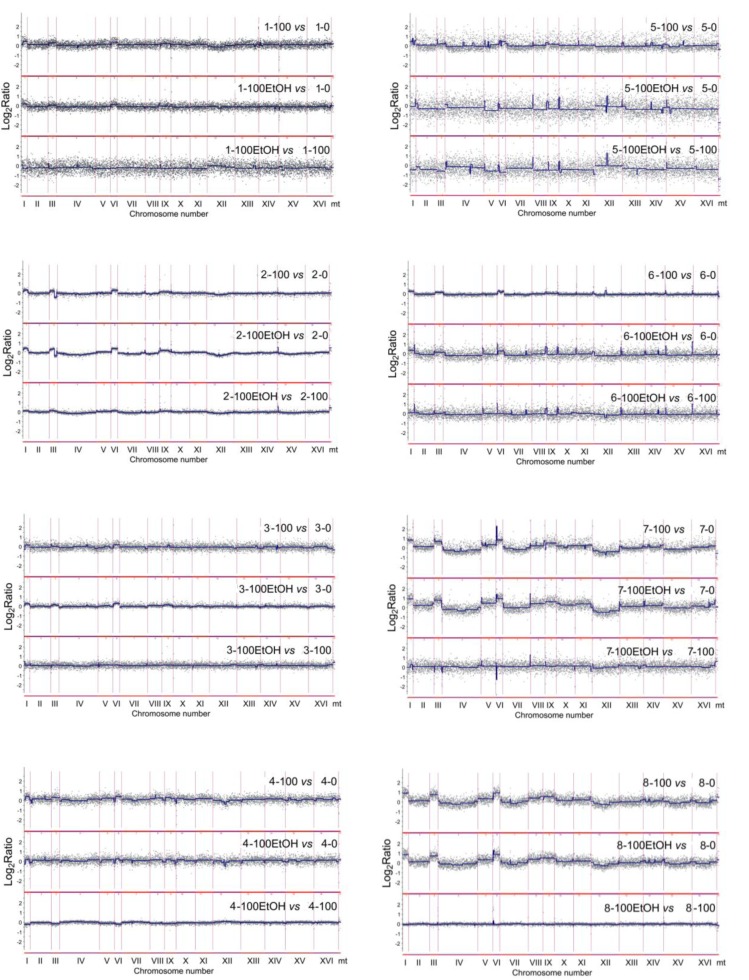
Generation- and ethanol-mediated changes in chromosome level using array-CGH Log_2_ ratios are shown. 0, control conditions; 100, 100 generations; 100EtOH, 100 generations in the presence of 5% EtOH. To obtain final CGH profiles, the data for each strain (1 to 8) were compared to control conditions of each strain. Moreover, comparison was made between passages in the absence and in the presence of ethanol.

According to array-CGH profiles, the most evident diversity in the gene copy number was revealed within subtelomeric regions in almost all analyzed chromosomes and within short intrachromosomal regions of chromosomes VIII, IX and XII (Figure 4 and Figure 5). Additionally, array-CGH profiles were used to estimate the level of similarity (relatedness) between wine strains on the basis of observed genomic diversity (Figure 4B). Strains 1 and 2, strains 3 and 5 and strains 7 and 8 were grouped together, whereas the most divergent strain was strain 4 with its own category (Figure 4B). As it is widely accepted that industrial yeasts are genomically diverse, we then decided to discriminate between genomic differences observed at control conditions and after passages with and without ethanol (Figure 6).

To focus on generation- and ethanol-mediated changes, array-CGH profiles during passages with and without ethanol were compared with array-CGH profiles at control conditions (Figure 6). Moreover, ethanol-associated array-CGH profiles were compared with array-CGH profiles during passages without ethanol (Figure 6). In general, changes in chromosome dosage observed at control conditions were augmented during passages (Figure 4 and Figure 6). In strain 1, the gains of chromosomes I, III, V and VI were propagated during passages both with and without ethanol (Figure 6). Some minor fluctuations in the levels of chromosomes II, IV, VIII, XII and XVI were also shown (Figure 6). Strain 2 is characterized by the duplication of a half of chromosome XVI at control conditions that is also observed during passages (Figure 4). The gains of chromosomes I and VI, and gross rearrangements within chromosome III were noticed during passages (Figure 6). In strain 3, the gains of chromosomes I, III, VI and IX were observed that were more accented during passages with ethanol than during passages without ethanol (Figure 6). The same changes in chromosome dosage were shown in strain 4 but the effects were more evident during passages in the absence of ethanol than in the presence of ethanol (Figure 6). Strains 5 and 6 were characterized by permanent loss and gain of chromosome IX, respectively (Figure 4). After 100 generations in the presence of ethanol, the loss of chromosomes I, III, V and VI and gain of chromosome XII were observed in strain 5, whereas in the case of strain 6, ethanol-induced gain of chromosomes I, III and VI were shown that was also noticed during passages without ethanol in strain 6 (Figure 4 and Figure 6). In strains 7 and 8, initial loss of chromosomes I, III, V, VI and IX was changed into the gain of chromosomes I, III, V, VI and IX during passages (Figure 4 and Figure 6). The effects observed for chromosomes I, III and VI were the most accented (Figure 6). Initial gain of chromosome XII was balanced during passages in strain 7 (Figure 4 and Figure 6). Array-CGH profiles of strains 7 and 8 were more variable than other analyzed array-CGH profiles that may indicate increased incidence of aneuploidy events in strains 7 and 8 (Figure 6). Taken together, the most frequently observed genomic change was the gain of chromosomes I, III and VI during passages (Figure 4 and Figure 6).

### Generation- and ethanol-mediated gene ontology overrepresentation profiles

**Figure 7:**
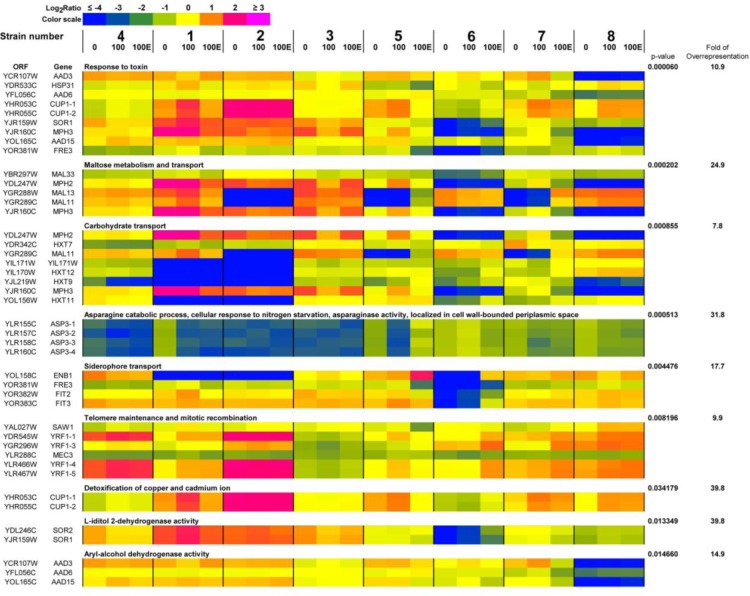
A heat map generated from array-CGH data Functional categories overrepresented in the group of genes that were the most divergent among analyzed strains are shown. The strains were ordered according to the result of clustering analysis (Figure 4B) and the selected genes were grouped according to their functional assignment. Positive and negative log_2_ ratio values represent higher and lower than average abundance of the gene, as determined by array-CGH analysis. 0, control conditions; 100, 100 generations; 100E, 100 generations in the presence of 5% EtOH.

The genes that were most divergent according to array-CGH-based analysis were then subjected to gene ontology overrepresentation analysis (Figure 7).

Nine functional categories overrepresented in the group of selected genes were revealed, namely 1) response to toxin, 2) maltose metabolism and transport, 3) carbohydrate transport, 4) asparagine catabolic process, cellular response to nitrogen starvation, asparaginase activity, localized in cell wall-bounded periplasmic space, 5) siderophore transport, 6) telomere maintenance and mitotic recombination, 7) detoxification of copper and cadmium ion, 8) L-iditol 2-dehydrogenase activity and 9) aryl-alcohol dehydrogenase activity (*p* < 0.05) and are presented as a heat map in Figure 7. In general, changes in gene dosage were more accented between wine strains analyzed than between passages and control conditions (Figure 7). Strain-dependent variability in the dosage of genes encoding hexose and maltose transporters and proteins involved in stress responses may reflect differences in fermentation performance and sugar utilization of commercially available wine yeasts used in this study. Passage-mediated gain of genes responsible for telomere maintenance (*YRF1* genes) and copper and cadmium detoxification (*CUP1* genes) was shown in strains 1, 4, 5, 6, 7 and 8 (Figure 7). In general, observed effects were similar during passages with and without ethanol (Figure 7). A heat map generated from array-CGH data reflecting the variability in the gene copy number of the whole genome of wine strains analyzed is also presented in Supplementary Material.

### Sirtuins are upregulated during passages

**Figure 8:**
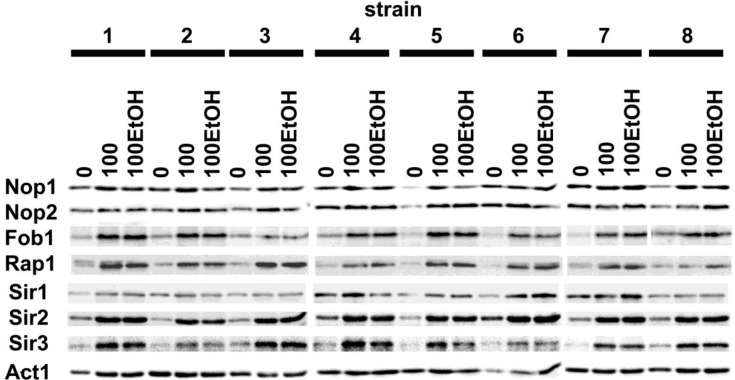
Generation- and ethanol-mediated changes in the levels of selected proteins Western blot analysis of Nop1, Nop2, Fob1, Rap1, Sir1, Sir2 and Sir3 contents. Anti-Act1 antibody served as a loading control. Lanes 0, control conditions; lanes 100, 100 generations; lanes 100EtOH, 100 generations in the presence of 5% EtOH.

Generation- and ethanol-induced increase in the protein levels of sirtuins, namely Sir1p, Sir2p and Sir3p was revealed (Figure 8).

Elevated expression of sirtuins was accompanied by increased protein level of Rap1, DNA-binding transcription regulator that interacts with Sir complex (Figure 8). Moreover, the levels of three nucleolar proteins, namely Fob1 and to a lesser degree Nop1 and Nop2 were also upregulated during passages in the presence and in the absence of ethanol (Figure 8).

### Generation- and ethanol-mediated changes at rDNA and in nucleolus state

**Figure 9:**
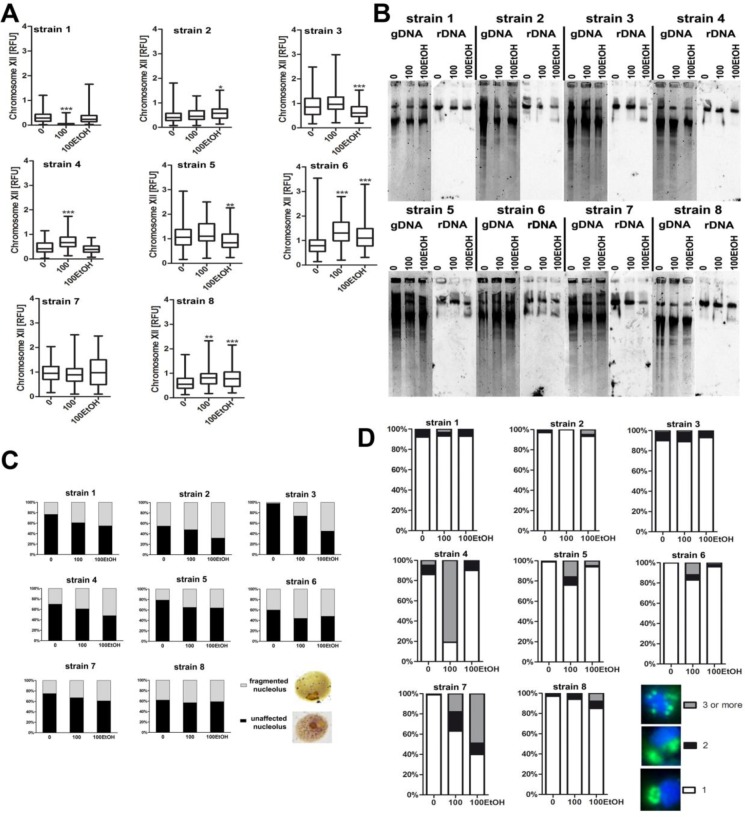
Generation- and ethanol-mediated changes in rDNA and nucleolus state **A.** Analysis of rDNA content. rDNA was visualized using WCPP specific to chromosome XII that contains rDNA locus in yeast. Fluorescence signals of chromosome XII were quantified using ImageJ software. The integrated fluorescence density is presented in relative fluorescence units (RFUs). 0, control conditions; 100, 100 generations; 100EtOH, 100 generations in the presence of 5% EtOH. Box and whisker plots are shown, *n* = 100. ^***^p < 0.001, ^**^p < 0.01, ^*^p < 0.05 compared to the standard growth conditions (ANOVA and Dunnett's *a posteriori* test). **B.** Southern blot analysis of the rDNA length. gDNA, genomic DNA after digestion. **C.** Silver staining of nucleolar organizer region-based analysis of nucleolus fragmentation. Fragmented nucleoli were scored [%]. The typical micrographs are shown (right). **D**. Analysis of chromosome XII signals using fluorescence *in situ* hybridization and whole chromosome painting probe (WCPP). Chromosome XII-specific signals were scored in 100 nuclei and presented as a percentage, namely three signal categories were considered: 1, 2 and 3 and more signals, *n* = 100. The typical micrographs are shown (right). The cells were labeled with FITC to detect chromosome XII-specific signals (green). DNA was visualized using DAPI staining (blue).

As passages affected the levels of nucleolar proteins (Figure 8), we then investigated rDNA and nucleolus states (Figure 9).

Except of strain 7, rDNA levels were altered during passages of wine yeasts, but not clear-cut relationship was noticed. An increase as well as a decrease in the levels of rDNA were shown (Figure 9A). In contrast, ethanol-mediated decrease in the length of rDNA was shown in strains 2, 3, 5 and 8 (Figure 9B). In all strains analyzed, nucleolus fragmentation was observed during passages in the presence and in the absence of ethanol (Figure 9C) that was accompanied by increased signals of chromosome XII that contains rDNA locus in strains 4, 5, 6, 7 and 8 (Figure 9D).

## DISCUSSION

In the present study, we found that chronic mild ethanol stress induced reactive oxygen species (ROS) and superoxide signaling and the upregulation of sirtuin proteins that promoted cell growth during passages. Industrial yeast cells also adapted to ethanol by changes in the dosage of genes involved in the telomere maintenance and ion detoxification.

It is widely accepted that the free radical theory of aging has some limitations and there are evidences that some experimental manipulations may promote healthspan and longevity by inducing hormesis effects in association with increased ROS levels in different model organisms and cell lines [21–31]. Both hydrogen peroxide and superoxide anions have been implicated in hormesis effects that promote longevity [26]. It has been reported that hydrogen peroxide extended chronological lifespan in the budding yeast as a response to caloric restriction or inactivation of catalases and in cells exposed to low levels of hydrogen peroxide [32]. Interestingly, the longevity phenotype of catalase mutants was accompanied by elevated damage in the form of protein carbonyls and lipofuscin [32]. Low levels of hydrogen peroxide also promoted replicative lifespan of human skin keratinocytes and prevented against telomere shortening [33]. It is postulated that lifespan-extending effect of hydrogen peroxide is mediated by hydrogen peroxide-induced superoxide dismutase (SOD) activity that inhibits the accumulation of superoxide anions in chronologically aging yeast cells [32]. In contrast, compromised TOR signaling extended yeast chronological lifespan by increased superoxide levels during yeast exponential growth that reduced ROS in post-diauxic and stationary phases [34]. The same effect was achieved by the treatment with superoxide-generating compound menadione and mitochondrial ROS signaling was suggested to be an important mechanism of longevity regulation [34]. Perhaps, ethanol-induced ROS and superoxide signals (this study) also promoted growth rate during passages when industrial yeast cells were cultured for 100 generations that was accompanied by increased protein levels of sirtuins, namely Sir1, Sir2 and Sir3, and DNA-binding transcription regulator Rap1. The yeast silent information regulator (Sir) protein complex has been implicated in transcriptional silencing and suppression of recombination at telomeres, silent mating-type loci and ribosomal DNA that may regulate the repair of DNA double-strand breaks, mitotic cell cycle, meiosis and aging [35]. Indeed, Sir proteins have been already described as regulators of longevity in the budding yeast and Sir2, a highly conserved NAD-dependent histone-deacetylase, also modulated the lifespan of worms and flies and metabolic health in mammals [36–40]. The replicative lifespan of *sir3* and *sir4* yeast mutants was found to be short that was due to the simultaneous expression of *a* and α mating-type information and elevated rate of rDNA recombination, likely increasing the rate of formation of extrachromosomal rDNA circles (ERCs) [36]. Thus, Sir2/3/4 complex was suggested to act indirectly to extend replicative lifespan by repressing transcription at *HML* and *HMR* [36]. Sir2p also acted directly to suppress ERC formation by inhibiting homologous recombination at a blocked replication fork in the rDNA and the short replicative lifespan of the *sir2* cells cannot be altered by deleting the *HM* loci [36]. Sir2p is thought to be a limiting component in promoting yeast longevity, because increasing the *SIR2* gene dosage extended replicative lifespan in wild-type cells [36]. Overexpression of *SIR2* also extended chronological lifespan and reduced acetate production during winemaking that indicated that Sir2p is a noteworthy factor for the improvement of alcoholic fermentation [41]. The acidification of the culture medium (i.e. acetic acid accumulation) is a well-recognized factor that limits survival and chronological lifespan of both vineyard and laboratory yeast cells [42, 43]. Interestingly, yeast-like chronological senescence has also been reported in mammalian cells [44]. However, the loss of replicative viability in a stationary culture of mammalian cells is caused by elevated lactate production [44].

More recently, the role of epigenetic silencing in mitochondrial ROS-induced longevity has been addressed [45]. A mitochondria-to-nucleus signaling pathway has been revealed in which the DNA damage kinase Tel1p transduces a mitochondrial ROS signal to activate Rad53p, which in turn regulates the ability of the histone demethylase Rph1p to bind subtelomeric chromatin [45]. The loss of Rph1p from subtelomeric chromatin elevates H3K36me3 and enhances binding of the silencing protein Sir3p to repress subtelomeric transcription [45]. Thus, mitochondria, epigenetics and telomere function have been shown to act together in the regulation of lifespan and ROS-induced Sir3p-mediated subtelomeric silencing may be considered an adaptation that extended chronological lifespan in yeast [45].

Wine yeast are highly specialized organisms that have adapted to cope with stressful fermentation environment, namely high osmolarity, reflecting increased sugar concentrations, high sulfite levels, anaerobiosis, acid stress, nutrient (nitrogen, lipids and vitamins) depletion and ethanol toxicity [46, 47]. The mechanisms of such human-enforced adaptive evolution may involve small-scale nucleotide changes (base insertions, deletions or substitutions), which alter protein structure, protein interactions or gene expression, large-scale genome rearrangements (chromosome duplications, translocations and aneuploidy), which alter gene expression through the modification of the genomic context, or copy number variations (CNV), which might alter the gene dosage, and contribute to the genetic and phenotypic diversity of wine yeasts [47, 48]. The best characterized adaptation is the resistance to sulfite, widely used preservative during winemaking, being a result of reciprocal translocation between chromosome VIII and XVI [49]. As genomes of industrial yeasts are dynamic and can undergo rearrangements, gene amplification and general genome instability in response to exposure to environmental stress [50], we analyzed then how non-cytotoxic concentration of ethanol can shape wine yeast genome during passages using genome-wide array-comparative genomic hybridization (array-CGH). The most pronounced differences in the gene copy number were shown within subtelomeric regions of all yeast chromosomes analyzed and the gains of chromosomes I, III and VI were the most frequently observed. Nine functional gene categories with statistically significant differences in the gene dosage were revealed, namely 1) response to toxin, 2) maltose metabolism and transport, 3) carbohydrate transport, 4) asparagine catabolic process, cellular response to nitrogen starvation, asparaginase activity, localized in cell wall-bounded periplasmic space, 5) siderophore transport, 6) telomere maintenance and mitotic recombination, 7) detoxification of copper and cadmium ion, 8) L-iditol 2-dehydrogenase activity and 9) aryl-alcohol dehydrogenase activity. In general, the effects were more strain-dependent than ethanol-dependent that may reflect different origin of wine strains purchased from multiple suppliers and also suggest that strains within wine group analyzed may differently respond to changing environments and may have diverse adaptation strategies. Ethanol-mediated gains of *YRF1* and *CUP1* genes were the most accented that may indicate the importance of telomere maintenance and ion detoxification for yeast cell adaptation to chronic mild ethanol treatment. The *YRF1* genes (*YRF1-1* to *YRF1-7*) are localized within the Y’ element of subtelomeric regions of different yeast chromosomes and encoded Y’ element ATP-dependent helicase (Y’-Help1, Y’-HELicase Protein 1) implicated in telomerase-independent telomere maintenance [51]. Perhaps, increased dosage of *YRF1* genes is a response to ethanol-induced loss of Y’ telomeric sequences during passages (this study). Y’-Help1, induced in telomerase deficient cells, is speculated to enhance homologous DNA recombination among Y’ elements and, as a consequence, induce Y’ amplification to prevent chromosomal loss and cell death [51]. More recently, we have also shown that increased *YRF1* gene copy number promoted genetic stability in distillery yeasts [52]. Cup1p is the major copper-activated metallothionein in yeast that binds copper and mediates resistance to high concentrations of copper and cadmium [53–55]. Moreover, Cup1p is also activated by Hsf1p in response to heat shock, glucose starvation and oxidative stress [56, 57]. Cup1p has been suggested to play a direct role in the cellular defense against oxidative stress by functioning as an antioxidant because yeast metallothionein may substitute for copper-zinc superoxide dismutase *in vivo* to protect cells from oxygen toxicity [58]. Perhaps, increased dosage of *CUP1* genes may also reflect a response to ethanol-induced ROS production during passages (this study). The relationships between ethanol and oxidative stress in laboratory and industrial yeasts have already been documented, especially the role of mitochondrial superoxide dismutase [15, 59–62]. Several transcription factors, namely Msn2p and Msn4p required for general stress response and Yap1p required for oxidative stress tolerance have also been implicated in the cellular response under ethanol stress as their binding motifs were found in the upstream sequence of many ethanol-induced genes [63]. However, it is worthwhile to remember that the acquisition of ethanol tolerance in yeast may involve interplay of many genes in complex networks at the genomic level and ethanol-responsive genes are associated with and overlapping with genes implicated in response to other environmental stress stimuli, such as heat shock or osmotic stress [63].

Ethanol also affected the nucleolus state during passages that indicates that ethanol is another stress stimulus being able to induce nucleolus-based response and confirms that nucleolus is a stress sensor in yeasts [64–66]. Ethanol caused an increase in the levels of nucleolar proteins, namely Fob1, Nop1 and Nop2, nucleolus fragmentation and changes in rDNA pools. Nop1 (ortholog of mammalian fibrillarin) and Nop2 (ortholog of mammalian p120), involved in pre-rRNA processing and ribosome biogenesis [67, 68], are histone glutamine methyltransferase that modifies H2A at Q105 and rRNA m5C methyltransferase that methylates cytosine at position 2870 of 25S rRNA, respectively [69, 70]. The effects of Nop1 and Nop2 proteins on nucleolus fragmentation have been documented [71–73]. Overexpression of *NOP2* gene resulted in nucleolus fragmentation [71]. Moreover, the upregulation of Nop2p and Nop1p was observed during chronological aging and passages in yeast, respectively, that was accompanied by nucleolus fragmentation [72, 73]. More recently, we have shown that industrial yeasts may adapt to changing environments by shifts in rDNA levels that acted as a regulatory mechanism to maintain chromosome homeostasis [73]. Thus, rDNA may also play a regulatory role during ethanol-induced genomic changes promoting more advantageous genetic features in wine yeast strains (this study). Moreover, ethanol-mediated Fob1 upregulation may also confirm the role of Fob1 in the modulation of rDNA stability and the promotion of cell survival [73, 74].

In conclusion, we show for the first time that chronic mild ethanol stress may promote an adaptive response in industrially relevant wine yeast strains that involves ROS and superoxide signaling, the upregulation of key longevity factors, namely sirtuins and the changes in chromosome dosage and gene copy number. Ethanol also affected the nucleolus state and the changes in rDNA pools may play a role in ROS-mediated cell proliferation that is in agreement with the role of rDNA and nucleolus in the maintenance of genome integrity and longevity [72, 73].

## MATERIALS AND METHODS

### Chemicals

All reagents were obtained from Sigma (Poznan, Poland) unless otherwise specified.

### Yeast strains and growth conditions

All wine yeast strains used in this work are listed in Table 1.

Yeast from one single colony was grown either on liquid YPD medium (1% w/v Difco Yeast Extract, 2% w/v Difco Yeast Bacto-Peptone, 2% w/v dextrose) or on solid YPD medium containing 2% w/v Difco Bacto-agar, at 28°C. Cells were cultured for 100 generations in the presence or absence of a non-cytotoxic concentration of ethanol of 5%, namely cells were grown for 6.(6) generations before being diluted (1:100) into fresh YPD medium [73].

### Growth rate and cell viability

For the kinetics of growth assay [75], cells were washed, diluted, suspended in YPD medium and cultured at 28°C. Their growth was monitored turbidimetrically at 600 nm in a microplate reader every 2 h during a 8 h period. Cell viability was estimated with a LIVE/DEAD^®^ Yeast Viability Kit (Thermo Fisher Scientific, Poland) using the standard protocol according to the manufacturer's instructions as described elsewhere [72]. Briefly, cells were washed and stained with a mixture of FUN^®^ 1 and Calcofluor^®^ White M2R and inspected under an Olympus BX61 fluorescence microscope equipped with a DP72 CCD camera and Olympus CellF software. Typically, a total of 200 cells were used for the analysis.

### FACS-based ploidy analysis

The DNA content was measured *via* flow cytometry as previously described [52].

### Pulsed-field gel electrophoresis (PFGE)

Preparation of agarose-embedded yeast DNA and PFGE separation of yeast DNA were conducted as described elsewhere [76].

### Detection of telomeric Y’ sequences

Y’ element telomeric probe was obtained according to [77] with minor modifications. After standard PFGE separation, Y’ sequences within particular yeast chromosomes were detected using digoxigenin labeling, anti-digoxigenin antibody and alkaline phosphatase-based chemiluminescence [72].

### Array-based comparative genomic hybridization (array-CGH)

Genomic DNA was labeled with SureTag DNA Labeling Kit and either Cy3- or Cy5-dUTP as previously described [52]. Briefly, equal amounts of labeled DNA of tested and of the reference laboratory strain (BY4741) were combined and hybridized to Yeast (V2) Gene Expression Microarray, 8×15K using Oligo aCGH Hybridization Kit. All components were supplied by Agilent Technologies Inc. (Santa Clara, CA, USA) and all steps of the experiment were performed according to manufacturer's protocols. Following hybridization and washing, the slides were scanned using Axon GenePix 4000B. Feature extraction was conducted using GenePix Pro 6.1 and normalization using Acuity 4.0 (Molecular Devices, Sunnyvale, CA, USA). CGH profiles with superimposed piecewise regression plots to highlight aneuploidies, were generated using CGH-Explorer v3.2 [78]. The original CGH profiles obtained after the comparison of analyzed strains to BY4741 gave consistently high noise due most probably to genomic DNA sequence differences between BY4741 and wine strains that influenced the hybridization strength of individual probes. Therefore to obtain final CGH profiles, the data for each strain were compared to the average of all strains analyzed.

### Gene analysis after array-CGH

The analysis of over-representation of functional categories was performed using Cytoscape v. 2.8.2 with BiNGO v. 2.44 plug-in and hypergeometric test using Benjamini and Hochberg False Discovery Rate (FDR) correction and significance level of 0.05.

### Cluster analysis

The array-CGH data were subjected to complete linkage clustering with Cluster 3.0 software using Euclidean distance similarity metrics [79] as previously described [52].

### Comet assay

Yeast spheroplasts were obtained [76] and DNA double-strand breaks (DSBs) and DNA single-strand (SSBs) breaks were assessed by neutral and alkaline single-cell microgel electrophoresis (comet assay), respectively, as described elsewhere [80]. The percentage of tail DNA was used as a parameter of DNA damage.

### Oxidative stress parameters

Intracellular reactive oxygen species (ROS) and superoxide production were measured using 2′,7′-dichlorodihydrofluorescein diacetate (H_2_DCF-DA) and dihydroethidium, respectively, as described elsewhere [72]. Oxidative DNA damage as a level of 8-hydroxy-2′-deoxyguanosine (8-OHdG, 8-oxo-dG) was measured using Epigentek EpiQuik 8-OHdG DNA Damage Quantification Direct Kit (Gentaur, Poland) as previously described [52]. Nuclear protein carbonylation was evaluated using nucleus and DNP co-staining. Protein derivatization was conducted according to [81]. Fixed and derivatized cells were incubated with the primary antibody anti-DNP (1:200) (Abcam) and the secondary antibody conjugated to FITC (1:1000) (Thermo Fisher Scientific). DNA was visualized using DAPI staining. Digital cell images were captured with an Olympus BX61 fluorescence microscope equipped with a DP72 CCD camera and Olympus CellF software. To analyze the level of nuclear protein carbonylation, ImageJ software *http://rsbweb.nih.gov/ij/* was used. Briefly, the integrated fluorescence density (green channel) that is the sum of all pixel values within the marked area of each nucleus analyzed and equivalent to the product of the area and mean gray value was evaluated. The integrated fluorescence density is presented in relative fluorescence units (RFUs).

### Western blotting

For WB analysis, whole cell extracts were prepared according to [72]. The following primary antibodies were used: anti-Nop1p (1:400), anti-Nop2p (1:400), anti-Fob1p (1:200), anti-Rap1p (1:400), anti-Sir1p (1:200), anti-Sir2p (1:200), anti-Sir3p (1:200) and anti-Act1p (1:1000) (Santa Cruz, Abcam). The respective proteins were detected after incubation with one of the horseradish peroxidase-conjugated secondary antibodies (1:80000, 1:100000 or 1:125000) (Sigma). The chemiluminescence signals were detected with a Clarity^™^ Western ECL Blotting Substrate (Biorad) and a G:BOX imaging system (Syngene, Cambridge, UK).

### rDNA analyses

rDNA was detected using both Southern blotting using rDNA specific probe [72] and fluorescence *in situ* hybridization (FISH) using whole chromosome XII painting probe [82]. For Southern blotting-based analysis of rDNA length, rDNA specific signals were detected using digoxigenin labeling, anti-digoxigenin antibody and alkaline phosphatase-based chemiluminescence after DNA digestion with *BamH*I. For FISH, biotin-labeled chromosome XII-specific DNA was detected using Star^*^FISH^©^ Biotin Painting Kit-FITC Label (Cambio, UK). Chromosome XII-specific signals were counted and presented as a percentage of 100 total cell scores. Moreover, to analyze the nucleolar rDNA content (chromosome XII-specific signals), ImageJ software was used as described elsewhere [76].

### Nucleolus morphology

To visualize the nucleolus, silver staining of nucleolar organiser regions (AgNOR) was performed. Silver staining of nucleolar argyrophilic proteins was conducted according to [72]. A total of 100 cells were analyzed and their nucleolus morphological type was determined (unaffected or fragmented nucleolus) [%].

### Statistical analysis

The results represent the mean ± SD from at least three independent experiments. Statistical significance was assessed by 1-way ANOVA using GraphPad Prism 5, and with the Dunnett's multiple comparison test.

## SUPPLEMENTARY TABLES



## References

[1] Ding J, Huang X, Zhang L, Zhao N, Yang D, Zhang K (2009). Tolerance and stress response to ethanol in the yeast Saccharomyces cerevisiae. Appl Microbiol Biotechnol.

[2] Stanley D, Bandara A, Fraser S, Chambers PJ, Stanley GA (2010). The ethanol stress response and ethanol tolerance of Saccharomyces cerevisiae. J Appl Microbiol.

[3] Farrell AE, Plevin RJ, Turner BT, Jones AD, O'Hare M, Kammen DM (2006). Ethanol can contribute to energy and environmental goals. Science.

[4] Wang Y, Zhang S, Liu H, Zhang L, Yi C, Li H (2015). Changes and roles of membrane compositions in the adaptation of Saccharomyces cerevisiae to ethanol. J Basic Microbiol.

[5] You KM, Rosenfield CL, Knipple DC (2003). Ethanol tolerance in the yeast Saccharomyces cerevisiae is dependent on cellular oleic acid content. Appl Environ Microbiol.

[6] Swan TM, Watson K (1998). Stress tolerance in a yeast sterol auxotroph: role of ergosterol, heat shock proteins and trehalose. FEMS Microbiol Lett.

[7] Hu CK, Bai FW, An LJ (2005). Protein amino acid composition of plasma membranes affects membrane fluidity and thereby ethanol tolerance in a self-flocculating fusant of Schizosaccharomyces pombe and Saccharomyces cerevisiae. Sheng Wu Gong Cheng Xue Bao.

[8] Takagi H, Takaoka M, Kawaguchi A, Kubo Y (2005). Effect of L-proline on sake brewing and ethanol stress in Saccharomyces cerevisiae. Appl Environ Microbiol.

[9] Kelley MJ, Bailis AM, Henry SA, Carman GM (1988). Regulation of phospholipid biosynthesis in Saccharomyces cerevisiae by inositol. Inositol is an inhibitor of phosphatidylserine synthase activity. J Biol Chem.

[10] Furukawa K, Kitano H, Mizoguchi H, Hara S (2004). Effect of cellular inositol content on ethanol tolerance of Saccharomyces cerevisiae in sake brewing. J Biosci Bioeng.

[11] Piper PW (1995). The heat shock and ethanol stress responses of yeast exhibit extensive similarity and functional overlap. FEMS Microbiol Lett.

[12] Wang PM, Zheng DQ, Chi XQ, Li O, Qian CD, Liu TZ, Zhang XY, Du FG, Sun PY, Qu AM, Wu XC (2014). Relationship of trehalose accumulation with ethanol fermentation in industrial Saccharomyces cerevisiae yeast strains. Bioresour Technol.

[13] Betz C, Schlenstedt G, Bailer SM (2004). Asr1p, a novel yeast ring/PHD finger protein, signals alcohol stress to the nucleus. J Biol Chem.

[14] Izawa S, Ikeda K, Kita T, Inoue Y (2006). Asr1, an alcohol-responsive factor of Saccharomyces cerevisiae, is dispensable for alcoholic fermentation. Appl Microbiol Biotechnol.

[15] Alexandre H, Ansanay-Galeote V, Dequin S, Blondin B (2001). Global gene expression during short-term ethanol stress in Saccharomyces cerevisiae. FEBS Lett.

[16] van Voorst F, Houghton-Larsen J, Jonson L, Kielland-Brandt MC, Brandt A (2006). Genome-wide identification of genes required for growth of Saccharomyces cerevisiae under ethanol stress. Yeast.

[17] Martinez-Pastor MT, Marchler G, Schuller C, Marchler-Bauer A, Ruis H, Estruch F (1996). The Saccharomyces cerevisiae zinc finger proteins Msn2p and Msn4p are required for transcriptional induction through the stress response element (STRE). EMBO J.

[18] Schmitt AP, McEntee K (1996). Msn2p, a zinc finger DNA-binding protein, is the transcriptional activator of the multistress response in Saccharomyces cerevisiae. Proc Natl Acad Sci U S A.

[19] Gasch AP, Spellman PT, Kao CM, Carmel-Harel O, Eisen MB, Storz G, Botstein D, Brown PO (2000). Genomic expression programs in the response of yeast cells to environmental changes. Mol Biol Cell.

[19] Naumov GI, Naumova ES, Lantto RA, Louis EJ, Korhola M (1992). Genetic homology between Saccharomyces cerevisiae and its sibling species S. paradoxus and S. bayanus: electrophoretic karyotypes. Yeast.

[21] Hekimi S, Lapointe J, Wen Y (2011). Taking a “good” look at free radicals in the aging process. Trends Cell Biol.

[22] Lapointe J, Hekimi S (2010). When a theory of aging ages badly. Cell Mol Life Sci.

[23] Ristow M, Schmeisser S (2011). Extending life span by increasing oxidative stress. Free Radic Biol Med.

[24] Labunskyy VM, Gladyshev VN (2013). Role of reactive oxygen species-mediated signaling in aging. Antioxid Redox Signal.

[25] Maryanovich M, Gross A (2013). A ROS rheostat for cell fate regulation. Trends Cell Biol.

[26] Ludovico P, Burhans WC (2014). Reactive oxygen species, ageing and the hormesis police. FEMS Yeast Res.

[27] Ristow M, Zarse K (2010). How increased oxidative stress promotes longevity and metabolic health: The concept of mitochondrial hormesis (mitohormesis). Exp Gerontol.

[28] Lee SJ, Hwang AB, Kenyon C (2010). Inhibition of respiration extends C. elegans life span via reactive oxygen species that increase HIF-1 activity. Curr Biol.

[29] Schulz TJ, Zarse K, Voigt A, Urban N, Birringer M, Ristow M (2007). Glucose restriction extends Caenorhabditis elegans life span by inducing mitochondrial respiration and increasing oxidative stress. Cell Metab.

[30] Yang W, Hekimi S (2010). A mitochondrial superoxide signal triggers increased longevity in Caenorhabditis elegans. PLoS Biol.

[31] Zarse K, Schmeisser S, Groth M, Priebe S, Beuster G, Kuhlow D, Guthke R, Platzer M, Kahn CR, Ristow M (2012). Impaired insulin/IGF1 signaling extends life span by promoting mitochondrial L-proline catabolism to induce a transient ROS signal. Cell Metab.

[32] Mesquita A, Weinberger M, Silva A, Sampaio-Marques B, Almeida B, Leao C, Costa V, Rodrigues F, Burhans WC, Ludovico P (2010). Caloric restriction or catalase inactivation extends yeast chronological lifespan by inducing H2O2 and superoxide dismutase activity. Proc Natl Acad Sci U S A.

[33] Yokoo S, Furumoto K, Hiyama E, Miwa N (2004). Slow-down of age-dependent telomere shortening is executed in human skin keratinocytes by hormesis-like-effects of trace hydrogen peroxide or by anti-oxidative effects of pro-vitamin C in common concurrently with reduction of intracellular oxidative stress. J Cell Biochem.

[34] Pan Y, Schroeder EA, Ocampo A, Barrientos A, Shadel GS (2011). Regulation of yeast chronological life span by TORC1 via adaptive mitochondrial ROS signaling. Cell Metab.

[35] Guarente L (1999). Diverse and dynamic functions of the Sir silencing complex. Nat Genet.

[36] Kaeberlein M, McVey M, Guarente L (1999). The SIR2/3/4 complex and SIR2 alone promote longevity in Saccharomyces cerevisiae by two different mechanisms. Genes Dev.

[37] Fabrizio P, Gattazzo C, Battistella L, Wei M, Cheng C, McGrew K, Longo VD (2005). Sir2 blocks extreme life-span extension. Cell.

[38] Covington JD, Bajpeyi S (2015). The sirtuins: Markers of metabolic health. Mol Nutr Food Res.

[39] Blander G, Guarente L (2004). The Sir2 family of protein deacetylases. Annu Rev Biochem.

[40] Giblin W, Skinner ME, Lombard DB (2014). Sirtuins: guardians of mammalian healthspan. Trends Genet.

[41] Orozco H, Matallana E, Aranda A (2013). Genetic manipulation of longevity-related genes as a tool to regulate yeast life span and metabolite production during winemaking. Microb Cell Fact.

[42] Burtner CR, Murakami CJ, Kennedy BK, Kaeberlein M (2009). A molecular mechanism of chronological aging in yeast. Cell Cycle.

[43] Murakami CJ, Wall V, Basisty N, Kaeberlein M (2011). Composition and acidification of the culture medium influences chronological aging similarly in vineyard and laboratory yeast. PLoS One.

[44] Leontieva OV, Blagosklonny MV (2011). Yeast-like chronological senescence in mammalian cells: phenomenon, mechanism and pharmacological suppression. Aging (Albany NY).

[45] Schroeder EA, Raimundo N, Shadel GS (2013). Epigenetic silencing mediates mitochondria stress-induced longevity. Cell Metab.

[46] Querol A, Fernandez-Espinar MT, del Olmo M, Barrio E (2003). Adaptive evolution of wine yeast. Int J Food Microbiol.

[47] Marsit S, Dequin S (2015). Diversity and adaptive evolution of Saccharomyces wine yeast: a review. FEMS Yeast Res.

[48] Querol A, Bond U (2009). The complex and dynamic genomes of industrial yeasts. FEMS Microbiol Lett.

[49] Perez-Ortin JE, Querol A, Puig S, Barrio E (2002). Molecular characterization of a chromosomal rearrangement involved in the adaptive evolution of yeast strains. Genome Res.

[50] James TC, Usher J, Campbell S, Bond U (2008). Lager yeasts possess dynamic genomes that undergo rearrangements and gene amplification in response to stress. Curr Genet.

[51] Yamada M, Hayatsu N, Matsuura A, Ishikawa F (1998). Y’-Help1, a DNA helicase encoded by the yeast subtelomeric Y’ element, is induced in survivors defective for telomerase. J Biol Chem.

[52] Deregowska A, Skoneczny M, Adamczyk J, Kwiatkowska A, Rawska E, Skoneczna A, Lewinska A, Wnuk M (2015). Genome-wide array-CGH analysis reveals YRF1 gene copy number variation that modulates genetic stability in distillery yeasts. Oncotarget.

[53] Karin M, Najarian R, Haslinger A, Valenzuela P, Welch J, Fogel S (1984). Primary structure and transcription of an amplified genetic locus: the CUP1 locus of yeast. Proc Natl Acad Sci U S A.

[54] Winge DR, Nielson KB, Gray WR, Hamer DH (1985). Yeast metallothionein. Sequence and metal-binding properties. J Biol Chem.

[55] Ecker DJ, Butt TR, Sternberg EJ, Neeper MP, Debouck C, Gorman JA, Crooke ST (1986). Yeast metallothionein function in metal ion detoxification. J Biol Chem.

[56] Tamai KT, Liu X, Silar P, Sosinowski T, Thiele DJ (1994). Heat shock transcription factor activates yeast metallothionein gene expression in response to heat and glucose starvation via distinct signalling pathways. Mol Cell Biol.

[57] Liu XD, Thiele DJ (1996). Oxidative stress induced heat shock factor phosphorylation and HSF-dependent activation of yeast metallothionein gene transcription. Genes Dev.

[58] Tamai KT, Gralla EB, Ellerby LM, Valentine JS, Thiele DJ (1993). Yeast and mammalian metallothioneins functionally substitute for yeast copper-zinc superoxide dismutase. Proc Natl Acad Sci U S A.

[59] Costa V, Reis E, Quintanilha A, Moradas-Ferreira P (1993). Acquisition of ethanol tolerance in Saccharomyces cerevisiae: the key role of the mitochondrial superoxide dismutase. Arch Biochem Biophys.

[60] Costa V, Amorim MA, Reis E, Quintanilha A, Moradas-Ferreira P (1997). Mitochondrial superoxide dismutase is essential for ethanol tolerance of Saccharomyces cerevisiae in the post-diauxic phase. Microbiology.

[61] Du X, Takagi H (2007). N-Acetyltransferase Mpr1 confers ethanol tolerance on Saccharomyces cerevisiae by reducing reactive oxygen species. Appl Microbiol Biotechnol.

[62] Bleoanca I, Silva AR, Pimentel C, Rodrigues-Pousada C, Menezes Rde A (2013). Relationship between ethanol and oxidative stress in laboratory and brewing yeast strains. J Biosci Bioeng.

[63] Ma M, Liu ZL (2010). Mechanisms of ethanol tolerance in Saccharomyces cerevisiae. Appl Microbiol Biotechnol.

[64] Olson MO (2004). Sensing cellular stress: another new function for the nucleolus?. Sci STKE.

[65] Lewinska A, Wnuk M, Grzelak A, Bartosz G (2010). Nucleolus as an oxidative stress sensor in the yeast Saccharomyces cerevisiae. Redox Rep.

[66] Grummt I (2013). The nucleolus-guardian of cellular homeostasis and genome integrity. Chromosoma.

[67] Tollervey D, Lehtonen H, Carmo-Fonseca M, Hurt EC (1991). The small nucleolar RNP protein NOP1 (fibrillarin) is required for pre-rRNA processing in yeast. EMBO J.

[68] Hong B, Brockenbrough JS, Wu P, Aris JP (1997). Nop2p is required for pre-rRNA processing and 60S ribosome subunit synthesis in yeast. Mol Cell Biol.

[69] Tessarz P, Santos-Rosa H, Robson SC, Sylvestersen KB, Nelson CJ, Nielsen ML, Kouzarides T (2014). Glutamine methylation in histone H2A is an RNA-polymerase-I-dedicated modification. Nature.

[70] Sharma S, Yang J, Watzinger P, Kotter P, Entian KD (2013). Yeast Nop2 and Rcm1 methylate C2870 and C2278 of the 25S rRNA, respectively. Nucleic Acids Res.

[71] de Beus E, Brockenbrough JS, Hong B, Aris JP (1994). Yeast NOP2 encodes an essential nucleolar protein with homology to a human proliferation marker. J Cell Biol.

[72] Lewinska A, Miedziak B, Kulak K, Molon M, Wnuk M (2014). Links between nucleolar activity, rDNA stability, aneuploidy and chronological aging in the yeast Saccharomyces cerevisiae. Biogerontology.

[73] Deregowska A, Adamczyk J, Kwiatkowska A, Gurgul A, Skoneczny M, Skoneczna A, Szmatola T, Jasielczuk I, Magda M, Rawska E, Pabian S, Panek A (2015). Shifts in rDNA levels act as a genome buffer promoting chromosome homeostasis. Cell Cycle.

[74] Kobayashi T (2006). Strategies to maintain the stability of the ribosomal RNA gene repeats—collaboration of recombination, cohesion, and condensation. Genes Genet Syst.

[75] Lewinska A, Macierzynska E, Grzelak A, Bartosz G (2011). A genetic analysis of nitric oxide-mediated signaling during chronological aging in the yeast. Biogerontology.

[76] Lewinska A, Miedziak B, Wnuk M (2014). Assessment of yeast chromosome XII instability: single chromosome comet assay. Fungal Genet Biol.

[77] Romano GH, Harari Y, Yehuda T, Podhorzer A, Rubinstein L, Shamir R, Gottlieb A, Silberberg Y, Pe'er D, Ruppin E, Sharan R, Kupiec M (2013). Environmental stresses disrupt telomere length homeostasis. PLoS Genet.

[78] Lingjaerde OC, Baumbusch LO, Liestol K, Glad IK, Borresen-Dale AL (2005). CGH-Explorer: a program for analysis of array-CGH data. Bioinformatics.

[79] de Hoon MJ, Imoto S, Nolan J, Miyano S (2004). Open source clustering software. Bioinformatics.

[80] Dworak N, Wnuk M, Zebrowski J, Bartosz G, Lewinska A (2014). Genotoxic and mutagenic activity of diamond nanoparticles in human peripheral lymphocytes *in vitro*. Carbon.

[81] Lazarus RC, Buonora JE, Jacobowitz DM, Mueller GP (2015). Protein carbonylation after traumatic brain injury: cell specificity, regional susceptibility, and gender differences. Free Radic Biol Med.

[82] Wnuk M, Miedziak B, Kulak K, Panek A, Golec E, Deregowska A, Adamczyk J, Lewinska A (2015). Single-cell analysis of aneuploidy events using yeast whole chromosome painting probes (WCPPs). J Microbiol Methods.

